# *Drosophila* increase exploration after visually detecting predators

**DOI:** 10.1371/journal.pone.0180749

**Published:** 2017-07-26

**Authors:** Miguel de la Flor, Lijian Chen, Claire Manson-Bishop, Tzu-Chun Chu, Kathya Zamora, Danielle Robbins, Gemunu Gunaratne, Gregg Roman

**Affiliations:** 1 Department of Biology and Biochemistry, University of Houston, Houston, TX, United States of America; 2 Biology of Behavior Institute, University of Houston, TX, United States of America; 3 Department of Physics, University of Houston, Houston, TX, United States of America; 4 Department of Biology, University of Mississippi, University MS, United States of America; University of Dayton, UNITED STATES

## Abstract

Novel stimuli elicit behaviors that are collectively known as specific exploration. These behaviors allow the animal to become more familiar with the novel objects within its environment. Specific exploration is frequently suppressed by defensive reactions to predator cues. Herein, we examine if this suppression occurs in *Drosophila melanogaster* by measuring the response of these flies to wild harvested predators. The flies used in our experiments have been cultured and had not lived under predator threat for multiple decades. In a circular arena with centrally-caged predators, wild type *Drosophila* actively avoided the pantropical jumping spider, *Plexippus paykulli*, and the Texas unicorn mantis, *Phyllovates chlorophaena*, indicating an innate defensive reaction to these predators. Interestingly, wild type *Drosophila* males also avoided a centrally-caged mock spider, and the avoidance of the mock spider became exaggerated when it was made to move within the cage. Visually impaired *Drosophila* failed to detect and avoid the *Plexippus paykulli* and the moving mock spider, while the broadly anosmic *orco*^*2*^ mutants were fully capable of detecting and avoiding *Plexippus paykulli*, indicating that these flies principally relied upon vison to perceive the predator stimuli. During early exploration of the arena, exploratory activity increased in the presence of *Plexippus paykulli* and the moving mock spider. The elevated activity induced by *Plexippus paykulli* disappeared after the fly had finished exploring, suggesting the flies were capable of habituating the predator cues. Taken together, these results indicate that despite being isolated from predators for decades *Drosophila* will visually detect these predators, retain innate defensive behaviors, respond by increasing exploratory activity in the arena rather than suppressing activity, and may habituate to normal predator cues.

## Introduction

Exploratory behaviors allow animals to gather information about their environment [1, 2]. These behaviors can be classified by how the animal explores its surroundings as well as categorized by the underlying motivational drive to explore. Specific exploration is motivated by novelty or a lack of information about the direct environment, and hence is driven by curiosity [1, 3]. Specific exploration is frequently accomplished through locomotor exploration which occurs when the animal moves to explore its environment [1]. The drive to explore novel features in the environment can be compelling in many species, superseding hunger, thirst, and even escape from predatory danger [4–6]. However, defensive reactions due to anxiogenic stimuli, which include predatory threats, can also strongly influence specific and locomotor exploration of novel environments in some species [7–9].

Many prey species display innate defensive reactions to predatory threats that depend on environmental cues like escape availability, predator type and predator proximity. For example, in the presence of bearded dragons (*Pogona vitticpes*) naïve adult crickets (*Gryllus texenis*) immediately seek shelter in the covered arms of a plus maze, whereas in the presence of a mock predator the crickets initially freeze before escaping suggesting innate differential responses to perceived predatory threats [10]. Different species of rodents will characteristically freeze or flee depending on the proximity and behavior of the predator, frequently swapping between the two defensive reactions in response to the predator’s behavior [11–13]. The perception of a specific threat threshold may be required for the initiation of escape behavior [14].

The perception of threat may also be changed through experience [15]. Habituation is a form of learning, likely present in all animal taxa, that drives the progressive decrease in behavioral responsiveness to repeated non-informative stimuli [16, 17]. In some circumstances, prey animals are capable of habituating to predator cues, leading to reduced defensive reactions to those predators [18, 19]. This habituation may allow the animal to reduce the cost of attention and heightened vigilance, allowing it to resume other adaptive behaviors such as foraging.

To begin to develop a better understanding of how behavioral conflicts between exploration and defensive reactions are resolved, we have examined the effects of predators on exploration in *Drosophila melanogaster*. Motivated by a lack of information, *Drosophila* display strong specific exploration of a novel circular open field arena [20, 21]. During exploration, the fly visits and revisits discrete areas of the arena boundary, visually habituates the novelty of these areas and learns about its environment [21, 22]. Habituation results in a reduction of specific and locomotor exploratory behaviors as the arena is learned and a steady state level of spontaneous activity is reached [23]. Herein, we examine how the presence of predator cues changes the exploratory activity of *Drosophila melanogaster* within a circular arena.

## Materials and methods

### Fly stocks and husbandry

All *Drosophila* were reared on a mixture of cornmeal, sucrose and yeast agar in culture bottles incubated at 25°C and 60% relative humidity in a 12:12hr LD cycle. To prevent larval crowding, the culture bottles were seeded with approximately 20 flies, allowed to mate freely over a period of 3–5 days before being removed. For behavioral experiments, 1–4 day-old *Drosophila* were collected under light CO_2_ anesthetization into food vials at a density of 15–20 flies per vial the day before assaying. The wild type Canton-S flies have been cultured in the Roman lab for more than 15 years. The *w*^*1118*^ flies (FBal0018186) were outcrossed into our Canton-S stock for ten generations. The *norpA*^*7*^ mutants (FBst0005685) were obtained from the Bloomington Stock Center, and the *orco*^*2*^ mutants (FBal0190982) were a generous gift of Leslie Vosshall (Rockefeller University). Both the *norpA*^*7*^ and *orco*^*2*^ mutations have been outcrossed into the Roman lab Canton-S background for more than seven generations.

### Predators

A total of three predator species and one non-predator species were used to manipulate the behavior of *Drosophila melanogaster*. The pantropical jumping spiders, *Plexippus paykulli*, and the twin-flagged jumping spiders, *Anasaitis canosa*, were captured on the University of Houston campus. The Texas unicorn mantis nymphs, *Phyllovates chlorophaena* (~ 2–3 cm in length) were kindly loaned to us by Dayne Jordan (University of Houston). At least three different individual predators for each species were used in experiments. The jumping spiders and mantis nymphs were housed in the lab in quart size plastic containers containing soil, a moist tissue and a small branch. Each predator was maintained in the lab for at least two months and fed four times a week a diet composed mostly of live *Drosophila*. We also used milkweed bugs, *Oncopeltus fasciatus*, as a non-predator control, which were obtained from Carolina Biological Supply Company (Burlington, NC) and cultured according to the supplier’s recommendations. The non-predator milkweed bug is similar in size to the spider predators and frequently moves within the central cage. We used these milkweed bugs as a control for some non-predator specific stimuli that may also be anxiogenic for *Drosophila*.

One week prior to their use in experiments, a predator or a non-predator would be habituated to the arena conditions and to being handled. This habituation procedure was used to reduce the escape behaviors of the centrally caged predators and non-predator controls. After this procedure, we found the predators focused their attention on the *Drosophila* within the arena. During this habituation, the predators or non-predators were placed inside the cage with a fly placed in the outer chamber of the arena. Approximately every 20–30 minutes the fly was replaced with a new fly. The predator would be subjected to this habituation for four hours a day for five days. At the end of this habituation period, the predators would no longer display obvious attempts to escape from the central cage. These predators would then be used in experiments during the following week.

To generate reproducible models of a mock spider, we made a 3-D scan of a generic toy Halloween spider. We then printed this spider in 3-dimensions on a Dimension 1200 es Printer (Dimension, Inc. Eden, MN). The printed mock spider was 1.8 cm long from head to abdominal thorax. These spiders were painted black and allowed to dry for more than one week before being used in experiments. To generate a spinning movement in the mock spider, a small stir bar was affixed to the spider’s underside. The arena was placed on a stir plate and the spider was set to spin asymmetrically at approximately 65 revolutions per minute.

### Circular arena paradigm

Activity in the arenas is captured at 30 f/s using a standard video camera mounted with a Navitar 7000 macro lens [20, 24]. Each Plexiglas arena is 8.2 cm in diameter (University of Houston Physics machine shop, Houston, TX). A 4.5 cm diameter nitex-walled cage is centrally located within the arena. The arena and the nitex cage walls are both 0.7 cm in height. Clear polystyrene lids for the arenas were used to keep the fly and predators inside while allowing the visualization of the fly’s behavior for recording purposes. The alcove arenas are almost identical to the 8.2 cm diameter circular arenas with the central cage, but with a small alcove 1.2 cm wide and 1.5 cm deep was added to one side. The arenas are placed about 1.2 meters below the video camera, and are lit by overlapping fluorescent flood lights (23W, 5100 K) emitting approximately 1000 lux. Ethovision XT v5.1 or v8.5 (Noldus, Leesburg, VA) software was used to digitally partition the arena into zones and to record activity. The 4.5 cm diameter central cage area was subtracted so that activity of the predator is not recorded. A 1.85 cm middle zone proximal to the cage was defined and a 1.85 cm outer zone was also defined. Ethovision was used to record locomotor activity and in which zone flies spent most of the time (positional preference) during each trial. Fly behavior is assayed for 10 minutes at 22–23°C and the % time in a zone for each trial was calculated by Ethovision.

During each open field trial, a predator was placed in the central caged chamber. The fly was then placed in the outer chamber of the arena and the assay would start immediately. To mitigate possible positional biases caused by environmental cues, the initial placement of flies in the outer chamber alternated between one of four cardinal starting positions (North, South, East, West). After four 10 min trials with different flies, the predator was removed and the arena wiped down with water and 70% ethanol and aired out for at least five minutes before the next round of trials. Then four new assays with different flies without the predator would be run. After those four trials, the arena would be cleaned again and the predator would be placed back into the cage for the next series of four trials with new flies. This essentially left the predator in the cage for less than an hour, and then removed for approximately an hour. To model learning during exploration and habituation we calculated coverage as a function of activity and P++, where activity is cm/sec and P++ is the probability of forward motion. To calculate coverage, the arena boundary is digitally discretized into patches the width of the system’s noise threshold. The system tracks the number of times a fly visits each patch. Where a coverage of 1 indicates the fly has visited all patches at least once, a coverage of 2 indicates two visits per patch. The x,y coordinates from each trial were exported from Ethovision XT into custom MatLab (MathWorks, Inc. Natick, MA) scripts and analyzed for activity, P++ and coverage measurements as described previously [22, 23].

### Statistics

Neither the time spent in the middle zone nor the time spent in the cove, in the absence of a predator, were found to follow a normal distribution for Cantons-S flies using the Shapiro-Wilk test [25]. Comparisons between two groups (e.g., control vs. predator) were carried out using the Mann-Whitney two-tailed test [26]. The Kruskal-Wallis test was used for comparisons of more than two groups [27], followed by Dunn’s two-tailed test with Bonferonni adjustments for multiple comparisons [28]. These statistics were calculated using XLSTAT version 2013.5.02 (Addinsoft, NY, NY).

The changes in activity and P++ as a function of arena boundary coverage represent a highly dense data set that was too large for normal analysis of variance tests (>120,000 data points/experiment). To determine if the treatments produced significant differences in exploration, we determined the F statistic for exponential regressions for each experiment [29]. The changes in activity and P++ as a function of coverage for each treatment was fit to a simple function with three variables: *y* = *a* * (1 + *x*/*b*)^*c*^. We then determined the variance for this regression within each treatment for each experiment. A regression was also fit to the same function for the entire experiment to determine the between group variance. The value of F was then determined as the (between group variance/degrees of freedom)/ (sum of the within group variance/degrees of freedom). This analysis was carried out for the entire coverage, or for periods of early exploration (coverage from 0 to 2), intermediate exploration (coverage from 2 to 4), and late exploration to spontaneous activity (4 to the end).

## Results

### Drosophila avoid caged predators

**Fig 1:**
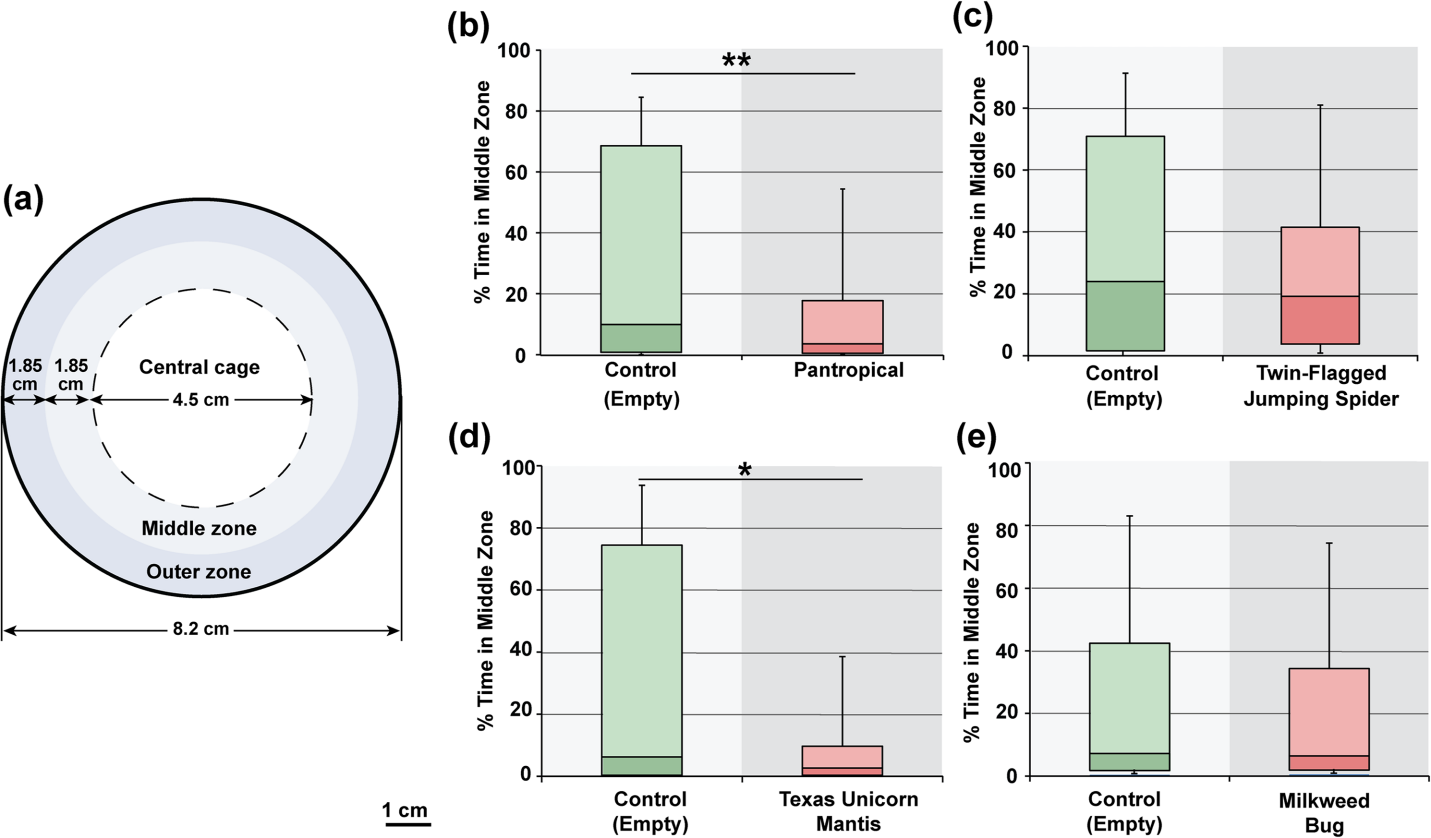
*Drosophila* predator avoidance in a circular arena. (a) A diagram of the 8.2 cm diameter arena with a central cage of 4.5 cm diameter to hold the predators is shown. To measure avoidance, the time spent in the middle zone adjacent to the central cage was measured. Wild type Canton-S flies spend significantly less time in the middle zone when (b) a pantropical jumping spider (*Plexippus paykulli*) is present. Canton-S did not significantly avoid (c) the twin-flagged jumping spider (*Anasaitis canosa*). (d) Wild type Canton-S males did avoid a Texas unicorn mantis (*Phyllovates chlorophaea*) nymph, (e) but not a non-predator milkweed bug (*Oncopeltus fasciatus*). ** p < 0.01, *p <0.05. The middle line of each box plot represents the median, while upper and lower box display the 3^rd^ and 2^nd^ quartile respectively. The whiskers show the 90% confidence intervals.

To measure the effect of predators on *Drosophila* exploratory activity, we first needed to find a predator that *Drosophila* detected and avoided in a circular arena. Relatively large circular arenas, like the one we used here, are advantageous for measuring exploration since they are highly symmetrical, have large surfaces that take time to learn, and lack corners that change the patterns of exploration [20, 21, 23]. The avoidance of the predator in this arena would indicate that wild type flies detected and recognized the predator as a threat. We measured this avoidance by comparing the flies’ positional preferences in an arena with different centrally caged predators to the positional preferences in the same arena without a predator. In a simple circular open field arena, *Drosophila* spend approximately 90% of time at the arena boundary, walking on and exploring the vertical wall [24, 30, 31]. Wall following behavior can be driven by directional persistence, or a failure to change directions when walking, but is also likely modified by an anxiety-like state of the *Drosophila* [24, 32]. For this experiment, we used an 8.2 cm diameter circular arena that contained a central nitex mesh-walled cage to house the predator (Fig 1A). When a central cage is added to this circular arena, the flies spend additional time exploring this added vertical surface, but they still spend the majority of time adjacent to arena boundary.

The following wild caught predators were used to measure predator avoidance in the arena with a central cage: the pantropical jumping spider, *Plexippus paykulli;* twin-flagged jumping spider, *Anasaitis canosa;* and the Texas unicorn mantis, *Phyllovates chlorophaena*. We also used the milkweed bug, *Oncopeltus fasciatus* as a non-predator control that was similar in size and shape to the predators, and moved within the central cage, but may have lacked stimuli specific to predators. The jumping spiders are hunting predators, and the mantis is an ambush predator. The twin-flagged jumping spiders were proximately 8 mm in length (cephalothorax and abdomen), while the pantropical jumping spiders were approximately 1.6 cm in length. Mantis nymphs that were between 2 and 3 cm in length were also used for these experiments. The predators were all housed inside the laboratory and fed a diet of *Drosophila* for more than two weeks prior to experimentation. When first placed into the caged chamber in the circular arena, the wild caught predators would frequently display vigorous attempts to move through the mesh cage, but with repeated exposures to the cage, these apparent escape behaviors habituated. Before beginning experiments with *Drosophila* each predator was allowed to habituate to the arena with an extended exposure over a one-week period. This procedure reduced the centrally caged animal’s escape behavior and allowed the predators to focus on the *Drosophila*. The predators were also starved for one day prior to being placed with an arena with a *Drosophila* to help them focus on the fly as potential prey. Consistent with their hunting strategies, the starved and habituated jumping spiders would frequently track the flies within the outer part of the arena (e.g., S1 Movie), while the mantids more frequently remained still with only occasional thrusts of the front tarsi towards the cage wall. The milkweed bug movements were frequent and appeared to be unconnected to the position of the fly.

Single Canton-S males were placed in the outer zone of the circular arena, with the indicated predator being placed in the central cage. Predator avoidance was measured as a significant decrease in the time spent in the middle zone, adjacent to the cage boundary, compared to the time spent in the absence of the predator. Interestingly, Canton-S flies appear to avoid some, but not all of the predators assayed. These flies would spend significantly less time adjacent to the cage in the presence of a pantropic jumping spider compared to when the cage was empty (Fig 1B; *U* = 6413.5, *N* = 126, *P* = 0.008). A similar avoidance was also found for the Texas unicorn mantis nymph (*U* = 5389, *N* = 95, *P* = 0.021), but not for the twin-flagged jumping spider (*U* = 8671.5, *N* = 128, *P* = 419), or the milkweed bug (*U* = 5825, *N* = 107, *P* = 0.808; Fig 1). Even though Canton-S flies did not statistically avoid the centrally caged twin-flagged jumping spider, they do consistently move away from uncaged twin-flagged jumping spiders, strongly suggesting the flies recognize these spiders as threats (S2 Movie). This avoidance of the uncaged spider further suggests that the time spent adjacent to the centrally caged predator is a conservative measure of predator avoidance.

The ability to detect predator avoidance by Canton-S flies in the circular arena was somewhat limited, in part because the flies spend a relatively small amount of time in the middle zone even in the absence of a predator. For each predator, there was a separate control group analyzed without the predator. We found that these control groups varied in the percent of time spent adjacent to the cage, with a range in medians from 24% in the twin-flagged jumping spider experiment to approximately 6% in the Texas unicorn mantis and 8% in the milkweed bug experiments (Fig 1). Each experiment was run for more than one week, and the different experiments shown in Fig 1 were separated by weeks. In the cases of the Texas unicorn mantis and the milkweed bug experiments, the relatively low amount of time the control groups spent in the middle zone could have reduced the ability to detect an avoidance of the caged insect.

**Fig 2:**
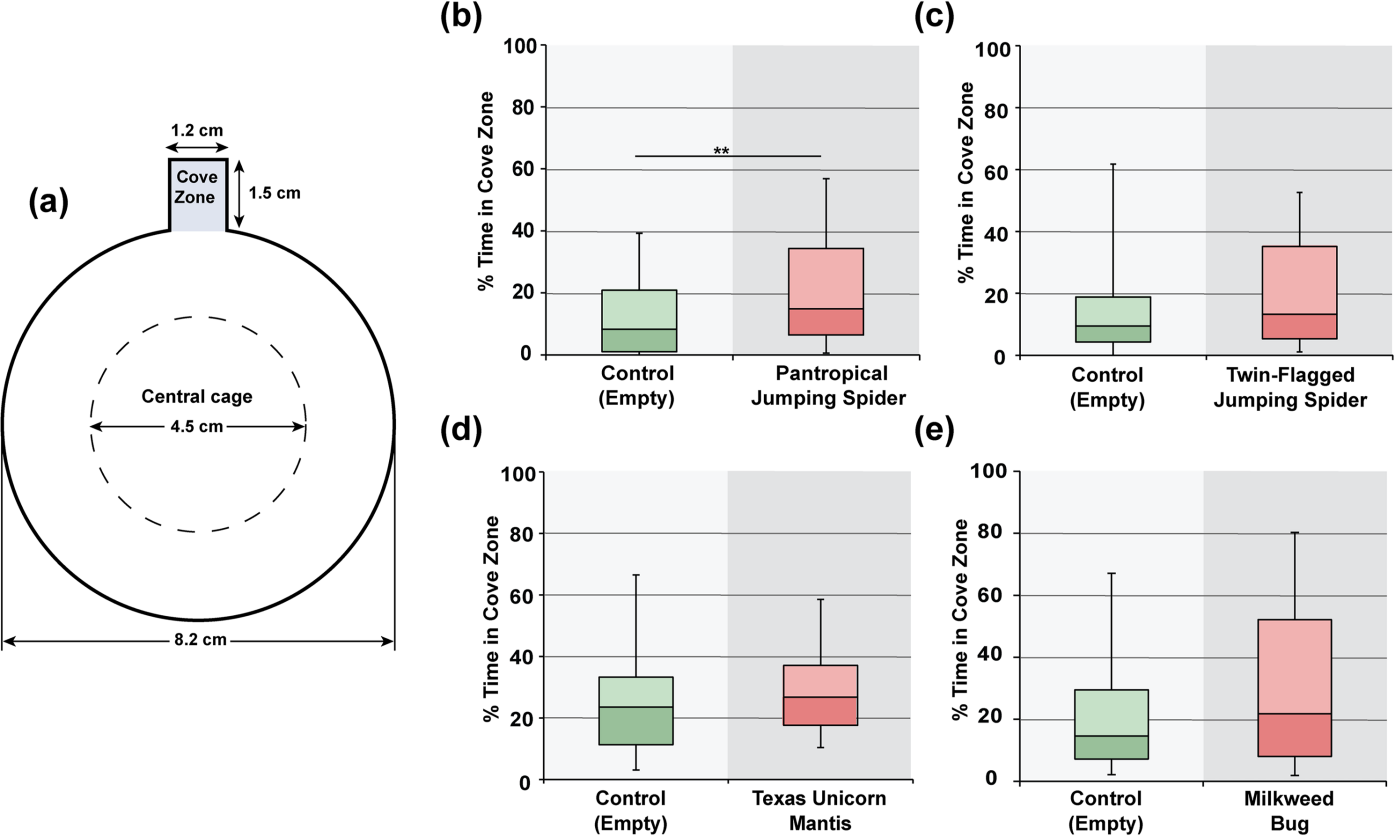
*Drosophila* predator avoidance in a circular arena with an alcove. A diagram of the 8.2 cm diameter arena with a central cage and an alcove is shown in (a). To measure avoidance in this arena, the % time spent in the cove zone during the 10 min assay is shown. Canton-S males spend significantly more time in the cove zone when (b) a pantropical jumping spider (*Plexippus paykulli*) is present. In this arena, Canton-S flies did not significantly avoid (c) the twin-flagged jumping spider (*Anasaitis canosa*), (d) the Texas unicorn mantis (*Phyllovates chlorophaea*) nymph, nor (e) the non-predator milkweed bug (*Oncopeltus fasciatus*). ** p < 0.01. The middle line of each box plot represents the median, while upper and lower box display the 3^rd^ and 2^nd^ quartile respectively. The whiskers show the 90% confidence intervals.

To verify the previous results of predator avoidance, we investigated a new measure using a circular arena with a small 1.2 X 1.5 cm^2^ alcove at one side (Fig 2A). We had previously found that Canton-S males will spend a relatively small, but significant time loitering within an identical alcove built into a circular arena [21]. Since the alcove provides an area that is farther from the predator, we hypothesized that the flies would increase the time in the alcove to maximize the distance from the predator. It is also possible that the flies increase their time spent in the alcove because it offers a sense of shelter. We found that Canton-S males spent significantly more time within the alcove in the presence of the pantropical jumping spider than when the predator is absent (Fig 2B; *U* = 5635, *N* = 95, P = 0.001), further demonstrating that wild type *Drosophila* detect and actively avoid this caged predator in a circular arena. The time spent in the cove zone did not increase significantly in the presence of the twin-flagged jumping spider (Fig 2C; *U* = 5088, *N* = 95, *P* = 0.129), Texas unicorn mantis (Fig 2E; *U* = 4076, *N* = 96, *P* = 0.167), or for the non-predator control milkweed bug (Fig 2F; *U* = 4325, *N* = 86, *P* = 0.076). These experiments with the cove arena provide further support that naïve wild type *Drosophila* detect the pantropical jumping spiders located in the central cage and find them aversive.

### Drosophila visually detect the caged pantropical jumping spider

The sensory systems used by *Drosophila* to detect the pantropical jumping spiders were next investigated using three sensory mutants. The *orco*^*2*^ mutation creates a loss of function at the *odorant receptor co-receptor* (*orco*) locus; *orco*^*2*^ flies are broadly anosmic, but otherwise healthy and long-lived [33, 34]. The *norpA*^*7*^ null mutation disrupts the visual phospholipase Cβ resulting in a complete loss of light-elicited photoreceptor potentials, and hence the *norpA*^*7*^ mutants are physiologically blind [35]. The *w*^*1118*^ loss-of-function mutation disrupts an ABC-like transporter that is necessary for the production of shielding eye pigments within the *Drosophila* compound eye [36]. The mutants remain phototactic, but have very poor visual acuity due to the absence of eye pigments and the resulting tangential activation of photoreceptor neurons [37].

**Fig 3:**
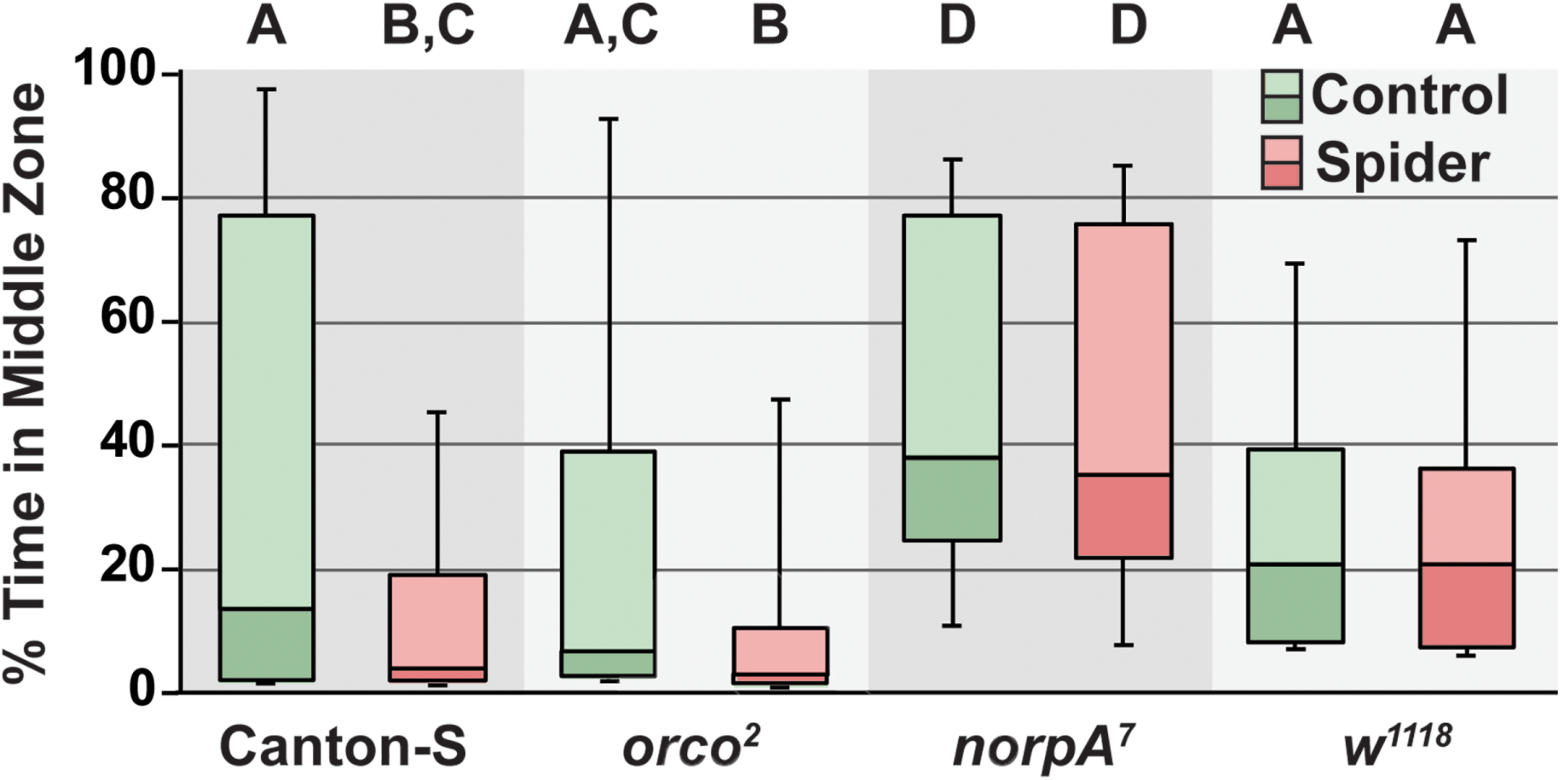
Visually impaired flies do not avoid the caged pantropical jumping spider. The mean % time spent in the middle zone adjacent to the central cage is shown for wild type and the indicated sensory mutants. Each genotype was examined with and without the presence of a pantropical jumping spider within the central cage. Significance differences are indicated by letters above each experimental groups. Groups sharing a letter are not different according to Dunn’s test (α = 0.0018). The middle line of each box plot represents the median, while upper and lower box display the 3^rd^ and 2^nd^ quartile respectively. The whiskers show the 90% confidence intervals.

We found significant differences in the avoidance of the pantropical jumping spider within the circular arena between two visually impaired mutant genotypes, the anosmic *orco*^*2*^, and wild type Canton-S (Fig 3; H_7_ = 219.61, N = 126 each group, P<0.0001). In this experiment the presence of the spider reduced the time spent adjacent to the cage for Canton-S (P<0.0001, α = 0.0018, Dunn’s multiple pairwise test with Bonferonni correction) and *orco*^*2*^ (P<0.0001), but not for *norpA*^*7*^ (P = 0.584) or *w*^*1118*^ (P = 0.957). In novel arenas, visually impaired flies display less wall-following behavior than normally sighted flies [20, 38], which can also be seen in the differences in the time spent in the middle zone of the blind *norpa*^*7*^ mutants compared to the normally-sighted Canton-S and *orco*^*2*^ mutants in the absence of the predator (Fig 3). The *norpA*^*7*^ flies in the absence of the spider also spent more time in the middle zone compared to the *w*^*1118*^ flies with no predator (Fig 3; P<0.0001). In this experiment, the times in the middle zone for the *w*^*1118*^ flies, with or without the spider, were not significantly different from the Canton-S or *orco*^*2*^ flies in the absence of this predator. The median time Canton-S spent in the middle zone in the presence of the caged predator was not significantly reduced compared to the time *orco*^*2*^ flies spent in the middle zone in the absence of predator, but there was trend in the expected direction (P = 0.008). In summary, two visually impaired fly genotypes failed to avoid the centrally caged predator, while a broadly anosmic genotype did avoid this spider; these results suggest that *Drosophila* rely principally on vision to detect the spiders.

**Fig 4:**
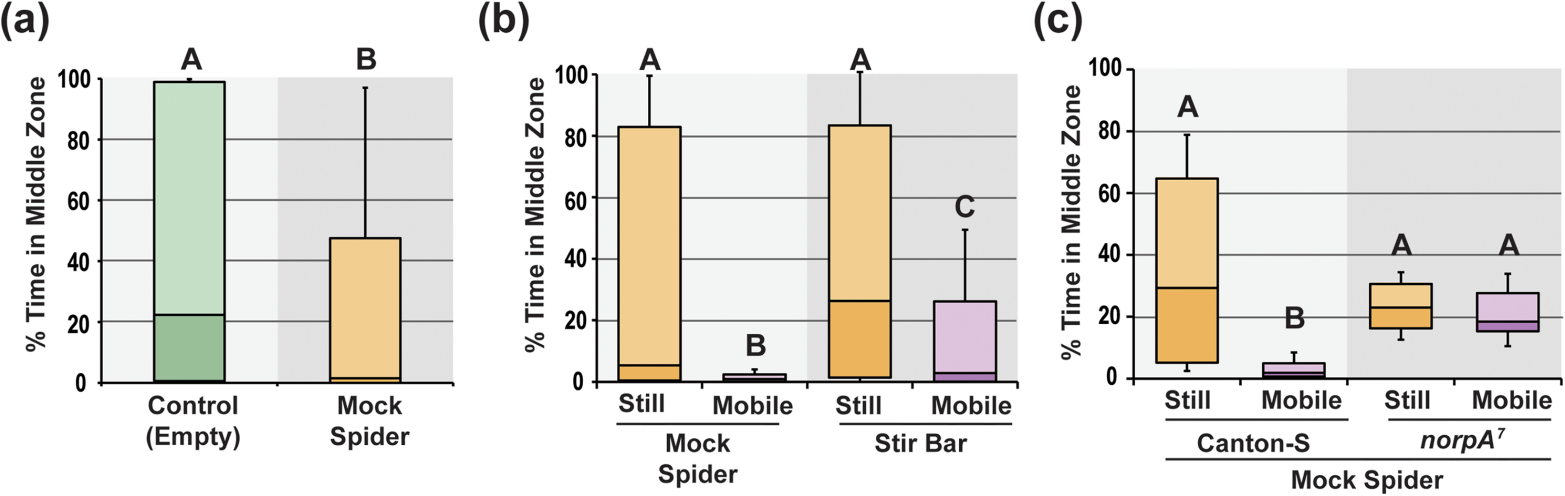
Vision is needed to avoid a moving mock-spider. The avoidance of the artificial predator is measured as the mean % time spent in the middle zone adjacent to the central cage. (a) The presence of an immobile mock-spider in the central cage significantly reduced the % time in the middle zone. (b) Canton-S flies are placed in the arena with either a centrally located mock-spider or a blackened stir bar. The avoidance of the middle zone by Canton-S is then measured with these objects immobile (still) or rotating (mobile). The movement of the mock spider and the stir bar generate a significantly greater avoidance of the middle zone than the still objects. The avoidance of the moving mock spider is even greater than the moving stir bar. (c) The blind *norpA*^*7*^ mutants do not avoid the moving mock spider, suggesting a visual detection of the moving mock-spider is required for this avoidance response. The middle point of each box plot represents the median, while upper and lower box display the 3^rd^ and 2^nd^ quartile respectively. The whiskers show the 90% confidence intervals. Groups with different letters situated above the column are significantly different from each other.

Visual cues may also be sufficient for predator avoidance behavior within the arena with a central cage. To test this hypothesis, we used mock plastic spiders located centrally. Several early studies on predator detection have shown the ability of vertebrates to display emotional affects to representations of predators [39–41]. The mock spiders that we used were black, had a spider’s general shape (head, abdomen and eight legs, S3 Movie), were somewhat larger than the pantropical jumping spider, but lacked odors, sounds, or other specific signaling behaviors associated with these spiders (e.g., seismic stomps [42]). Wild type Canton-S flies significantly avoided this caged mock spider in the circular open field arena (Fig 4A; *U* = 5793.5, *N* = 96, *P* = 0.002).

*Drosophila* strongly attend looming stimuli [43], and hence it was possible that movement may induce looming and make the mock spider even more aversive. To test this prediction, we added a stir bar to the mock spider’s abdomen and used a stir plate to induce an asymmetric spinning movement to test if this increased the avoidance response (Fig 4B; S3 Movie). In this experiment, a black stir bar (3 cm in length) was also used as a control for general movement effects of the spider and the seismic activity of the stir plate. We found that there were significant differences between the four groups in this experiment (*H*_3_ = 33.62, P < 0.0001). Canton-S flies spent significantly less time adjacent to the cage when the mock spider was moving compared to when the mock spider was motionless (Fig 4B; *W* = 5.498, *N* = 32, P = 0.001). In fact, Canton-S flies hardly approached the cage at all when the mock spider was spinning. The moving stir bar also generated a decreased time spent in the middle zone compared to the motionless stir bar (*W* = 4.140, *N* = 32, *P* = 0.018), however the moving spider also induced a significantly greater avoidance response compared to the moving stir bar (*W* = 4.3985, *N* = 32, *P* = 0.010). These results indicate that the moving mock spider and the moving stir bar are sufficient to generate a robust avoidance response in the open field arena, and that features of the mock spider’s movement generate an even greater avoidance response than the moving stir bar.

The robust avoidance of the moving mock spider may be due to the flies’ perception of several stimuli including vibrations in the arena generated by the stir plate, the sound of the rotating spider, air movement created by the rotations that passes through the nitex mesh of the cage wall, or perhaps even the visual detection of features found in the mock spider but missing in the stir bar, e.g., the spider’s uneven shape may indicate a predator looming. Since the blind *norpA*^*7*^ failed to detect and avoid the pantropical spider, we also examined the ability of these blind mutants to avoid the mock spider (Fig 4C). There were significant differences between the four groups in this experiment (*H*_3_ = 48.278, *N* = 32, *P* <0.001). Similar to the previous experiment, the wild type control flies spent less time in the middle zone adjacent to the moving mock spider than when the immobile mock spider was located within the central cage (*W* = 7.140, *N* = 32, *P* < 0.0001). Interestingly, the blind *norpA*^*7*^ mutants do not avoid the moving mock spiders as compared to the motionless mock spider (*W* = 1.595, *N* = 32, *P* = 0.672), strongly suggesting that this robust avoidance response is driven by the visual detection of stimuli from the moving mock spider (Fig 4C).

### The presence of a predator increases locomotor exploration

The avoidance of the pantropical jumping spider by Canton-S flies in both the simple circular and the alcove arenas suggests that *Drosophila* recognizes this predator as a threat. Predatory threats within a fly’s flight initiation distance may increase exploratory activity geared towards seeking escape from the arena, a type of extrinsic exploration. More distal predatory threats may elicit freezing-like behaviors [11, 44–46]. Freezing behaviors suppress locomotion, making the prey less obvious to the predator and allow the prey to focus on the predator’s behavior. To see if *Drosophila* display either of these two defensive behaviors, we measured the exploratory activity of Canton-S flies exposed to a pantropical jumping spider or the mock spider.

Locomotor exploration in *Drosophila* can be seen as an elevated activity when first placed within the novel arena [20, 21]. This elevated activity decays to a steady state spontaneous activity within a few minutes of locomotor exploration. As the fly explores, it visits each area of the arena boundary, providing it an opportunity to learn this location and relying heavily on visual cues to habituate the novelty [21, 24]. During the early exploration period, flies also display a very high probability for continued forward motion (P++), with low probabilities for stopping (P+0) or reversals (P+-) of direction [22, 24]. This initially high P++ may represent the goal-directed seeking of novel patches of the arena boundary [22]. In contrast, P+0, is the probability to initiate a pause in movement, and this measure increases as the fly becomes more familiar with its environment. To measure the opportunities for the fly to learn the arena boundary (training trails in this non-associative learning), we use the fly’s coverage, which is defined as the minimal number of visits to each area of the arena boundary [22]. As expected for measures of locomotor exploration, the activity and P++ of a fly attenuate as a function of arena coverage independent of the arena’s size [22, 23].

**Fig 5:**
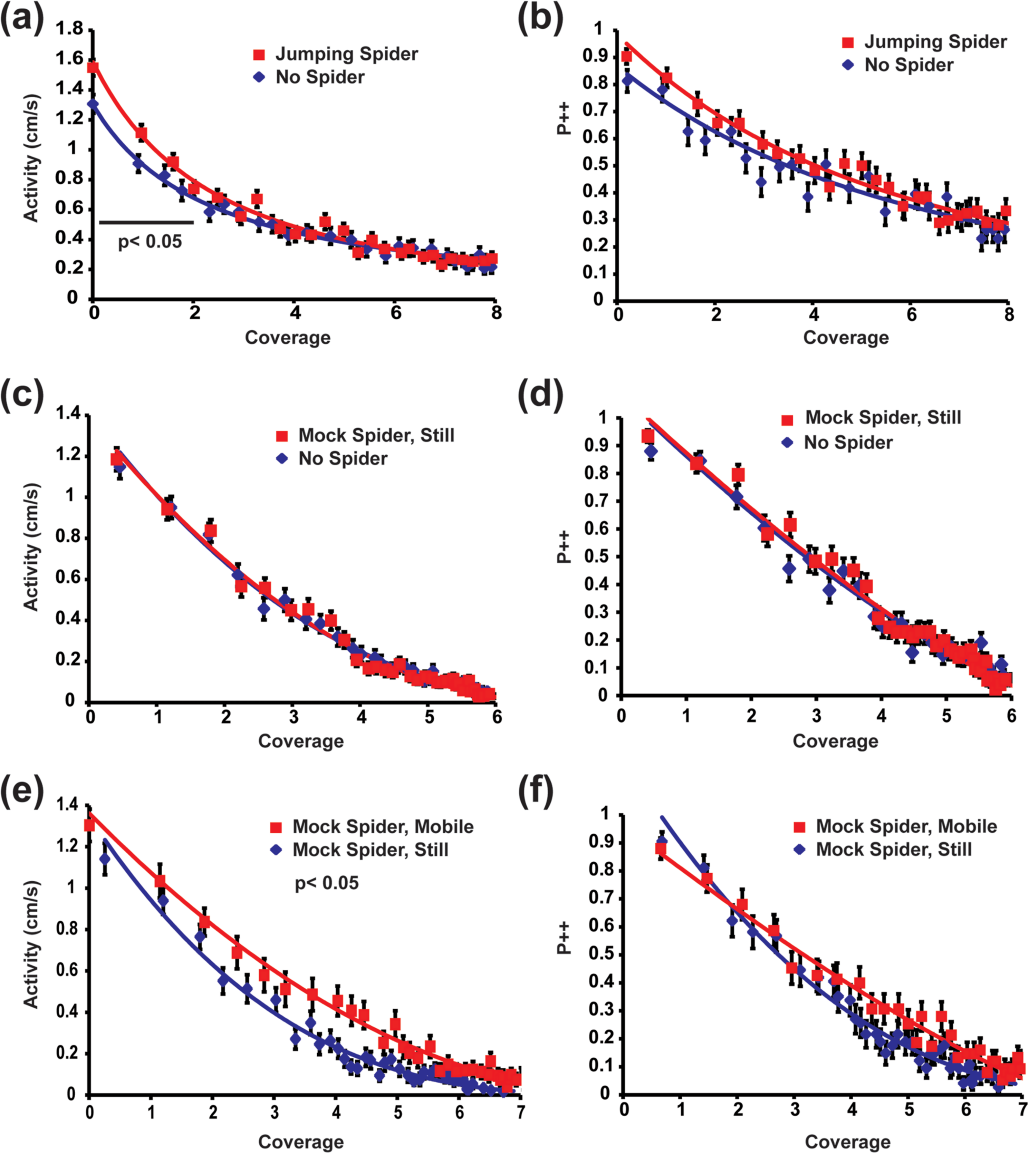
*Drosophila* increase activity in the presence of pantropical jumping spiders and moving mock spiders. The changes in activity of the fly (cm/sec) and the probability for continued forward motion (P++) as a function of the minimum number of visits to each discrete section of the arena boundary (coverage) is shown. Both activity and P++ decrease as the fly habituates to the novel arena boundary [22, 23]. (a) In the presence of the pantropical jumping spider, Canton-S males significantly increase their activity during the early exploration of the arena, coverage 1–2. (b) Canton-S flies do not alter their P++ in the presence of the pantropical jumping spider. (c) The activity or (d) the P++ of Canton-S flies in the presences or absence of a mock spider were not significantly different. (e) Canton-S males display significantly greater activity in the presence of a moving mock spider as compare to an immobile mock spider. (f) The moving mock spider does not significantly change the P++ of Canton-S flies compared to the still mock spider. Shown are the mean +/- standard error of the mean for each data point. The lines show the regressions fit to *y* = *a* * (1 + *x*/*b*)^*c*^ for each experimental group.

In the presence of a pantropical jumping spider, wild type flies displayed a significant increase in activity during the early phase of exploration, but the difference in P++ was not significant (Fig 5A and 5B). The significant increase in activity for the spider-exposed flies was found only during the initial coverage period of 0–2, when flies visited each area of the boundary less than twice (Fig 5A; F(1,205) = 4.56, p = 0.034). After the flies became more familiar with the arena, the effect of the spider on activity is no longer significant (Coverage 2–4, F(1,205) = 0.49, p = 0.483). The flies’ P++ in the presence of the spider trended slightly higher than the control flies P++ during the first period of coverage from 0–2, but they were not significantly different (Fig 5B; F(1,205) = 2.85, p = 0.092). The probability that the fly would initiate a stop (P+0) during the early coverage period of 0–2, was significantly lower in the presence of the spider compared to the control group (F(1,205) = 4.55, p = 0.034; data not shown). The effect of the spider on initiating stops (P+0), was not found after further exploration, during the coverage 2–4 (F(1,205) = 0.789, p = 0.375; data not shown). Overall, these data demonstrate an early effect of the spider’s presence with increased activity during the active exploration period, but no significant effects of the spider once the arena has become familiar. No evidence of any freezing behavior was detected; the flies moved more in the presence of the spider and P++ behavior was also not reduced.

Since the moving mock spider elicited from the Canton-S males a very strong avoidance response, we predicted that the effect of exposure on increased exploration would be at least as strong as the exposure to the pantropical jumping spider. The simple presence of the mock spider did not have a significant impact on activity (Fig 5C; coverage 1-end; F(1,238) = 0.079, p = 0.779) or P++ (Fig 5D; coverage 1-end; F(1,238) = 0.035, p = 0.851). A moving spider however, led to a significantly higher overall activity as compared to the motionless mock spider (Fig 5E; coverage 0-end, F(1,149) = 3.98, p = 0.047). Interestingly, the greatest difference in activity found between the still and mobile spider was not during the period of early exploration (coverage 0–2, F(1,149) = 2.04, p = 0.156), but later during the period of spontaneous activity (coverage 4-end, F(1,149) = 4.08, p = 0.045). The presence of the moving mock spider did not generate any significant difference in P++ compared to the control group (Fig 5F; coverage 0-end, F(1,149) = 1.33, p = 0.251).

## Discussion

Herein, we have examined the effect of natural and artificial predators on the exploratory behavior of *Drosophila melanogaster*. Canton-S flies detect and avoid both the pantropical jumping spiders and the Texas unicorn mantis nymphs in a circular arena. Since our flies have been kept in culture and under no predation threat for decades, the predator avoidance is clearly an innate defensive behavior. Canton-S flies demonstrate an even stronger avoidance of a mobile mock spider. These flies rely primarily on visual cues to detect the pantropical jumping spider and the moving mock spider. The flies’ responses to the pantropical jumping spider and the mock spider display some interesting differences. Wild type flies initially increase their activity in the presence of the pantropical jumping spider, but this activity undergoes habituation to normal levels of spontaneous activity as the fly explores the arena, whereas the activity in the presences of the moving mock spider remains higher throughout the experiment. Differences in the exigency of the mock spider threat vs. the pantropical spider threat are probably responsible for the exaggerated responses to the continually moving mock spider. It is likely that these increases in exploratory activity represent an anxiety-like response to perceived predatory threats.

The strongest natural predator avoidance we detected was to a hunting predator that frequently moved and tracked the position of the fly from within its central cage (S1 Movie), although we also detected significant avoidance of the Texas Unicorn Mantis, an ambush predator. These data indicate that naive, laboratory raised *Drosophila* can recognize both classes of predators as a threat. Although we didn’t detect avoidance of the caged twin-flagged jumping spider in our arena, *Drosophila* are generally sensitive to the presence of these predators. *Drosophila* adults run away from the approaches of an uncaged twin-flagged jumping spider within the open field arena (S2 Movie). Our inability to detect avoidance of these latter caged predators was likely due in part to the imperfect avoidance paradigm we used for these experiments. The circular arena with the centrally-caged predator was chosen for this study since this arena provides for strong exploration of the boundary, but this arrangement is not ideal for detecting avoidance of a centrally caged predator [20, 21]. The central cage allows the fly to continue exploring the arena boundary, and remain exposed at all sides to the predator. However, since flies spend a majority of time at the arena boundary [31, 38], and the general tendency to follow walls in an arena appears to be at least partially driven by an evolutionarily conserved anxiety-like process [32], there is not much statistical power to detect differences in wall following behavior. For example, the smaller size of the twin-flagged jumping spider within the cage would have generated a reduced looming threat, potentially making it less aversive. The larger pantropical jumping spider, with its greater profile, would generate a shorter flight initiation distance in a fly than the smaller twin-flagged jumping spider. This shorter flight initiation distance for the pantropical jumping spider would be more readily detected as avoidance in our arenas. It is possible that even if the twin-flagged jumping spider was detected as a threat, it may have only been detected by the fly when the spider was directly adjacent to the cage wall nearest the fly, and hence we would not have seen avoidance in our paradigm. It is also possible that *Drosophila* can just more quickly habituate the threat cues of the twin-flagged spider.

The role of vision in predator detection was demonstrated by the failure of two visually impaired genotypes to avoid the pantropical jumping spider. In another interesting study, *Drosophila melanogaster* were shown to visually learn about the presence of the parasitoid wasp *Leptopilina heterotoma* within the population, and respond by changes in oviposition substrate presence or through apoptosis of oocytes [47, 48]. *Drosophila* socially transmit information regarding the presence of these wasps using their wings; the broadly anosmic *orco*^*1*^ were able to learn about the presence of the wasps in the population, while the visually impaired *ninaB*^*P315*^ mutants could not learn [47]. In contrast, our experiments are with single flies that visually and directly detect the presence of the predators.

The specific visual stimuli of the spider that the flies detect as aversive/threatening in our experiments is not yet clear. There appeared to be differences in the strength of the avoidance responses to the predators, from weaker avoidances of the still mock spider to intermediate avoidances of the pantropical jumping spider and the moving stir bar to the strongest avoidances of the moving mock spider. While it is possible that a spider gestalt (a configuration of stimuli present in the spiders) is recognized as a threat to be avoided [39, 41], there may also be discrete stimuli that are additively effecting avoidance. The moving stir bar and the rotating mock spider in particular may have elicited a looming/collision threat [49–52]. The threat of collision combined with the irregular form of the mock spider could act as heterogeneous stimuli that when summed, release a stronger escape response [53]. In effect, since the flies display an amplified response to this unnaturally exaggerated stimuli, the moving mock spider may represent a super-normal predator stimulus [54, 55].

### Behavioral plasticity in response to a predator

Predators are believed to be robust agents for inducing fear or anxiety in many prey species [56–58]. The behavioral responses to predators depend on several factors including the degree of anxiety and fear induced in the prey, the presence and distance of refuge, distance from predator, predator size and predator movement [44–46, 59]. In the absence of shelter, prey animals will choose to either flee from a predator or freeze, frequently alternating between the two [9, 60]. In the presence of the pantropic jumping spider, wild type *Drosophila* initially show increased locomotor activity compared to flies in the absence of predators. This increased level of activity most likely represents *extrinsic exploration* for safety. As the fly explores the arena and learns there is neither escape nor shelter, the amount of activity decays to normal levels of spontaneous activity. In contrast, the moving mock spider continues to evoke increased activity even after the arena has been learned. Interestingly, we did not see any differences in the probability of directed movement (P++) in either the presence or absence of the spider, indicating that these flies did not display freezing behavior in the open field arena. Although many small rodents have adopted freezing as an effective defense against aerial and terrestrial predators [8, 60, 61], it is possible that freezing as a defensive response to a terrestrial predator may have little adaptive value for prey species that are capable of flying.

Canton-S flies were able to habituate to the presence of the caged pantropical jumping spider as well as to the arena’s novelty. Canton-S flies displayed heightened activity while exploring in the presence of the predator, but this heightened activity was no longer present once the flies finished exploring the arena, demonstrating that they were capable of habituating the predator stimuli. Habituation to predator specific stimuli has significant adaptive value as it allows animals to minimize the cost of escape behaviors in the face of predator false alarms [18, 62]. In this context, it is not surprising that the potential super-normal stimuli of the moving mock spider continued to drive higher levels of activity in Canton-S flies after the arena had been fully explored. Strong stimuli frequently take longer to habituate, or escape habituation entirely [16, 63, 64].

## Supporting information

S1 Movie A caged pantropical jumping spider tracking the position of wild type Canton-S *Drosophila melanogaster* within the open field arena. This spider was habituated to the arena before this experiment, and was not displaying active escape behaviors.
(MOV)

S2 Movie A Canton-S male avoids an uncaged twin-flagged jumping spider within an open field arena. This male fly was placed within an open field arena along with the spider. This spider was habituated to the arena prior to this movie.
(MOV)

S3 Movie A caged moving mock spider within an open field arena. This plastic spider was made to move by adding a small stir bar to its abdomen and then mounting the arena on a stir plate.
(MOV)
